# Finding the optimal regimen for short-term daily recombinant human erythropoietin treatment for blood-saving purpose in patients undergoing unilateral primary total hip arthroplasty: a double-blinded randomized placebo-controlled trial

**DOI:** 10.1186/s12891-022-05184-1

**Published:** 2022-03-12

**Authors:** Mingcheng Yuan, Qifeng Tao, Duan Wang, Haoyang Wang, Zongke Zhou

**Affiliations:** 1 Department of Orthopedics, West China Hospital/West China School of Medicine, Sichuan University, 37# Wuhou Guoxue Road, Chengdu, People’s Republic of China; 2 Department of Orthopedics, Panzhihua Municipal Central Hospital Panzhihua, Panzhihua, People’s Republic of China

**Keywords:** Total hip arthroplasty, Recombinant human erythropoietin, Blood-saving, Optimal regimen

## Abstract

**Purpose:**

To find the best short-term daily recombinant human erythropoietin (rhEPO)-based treatment protocols for blood-saving purpose in THA.

**Method:**

The patients were randomized to 1 of 3 interventions: Patients in group A received 10,000 IU (150 IU/kg) of subcutaneous rhEPO (1 ml) daily from 5 days preoperatively to 3 days postoperatively (9 doses in total); Patients in group B received 1 ml of subcutaneous normal saline daily from 5 days preoperatively to 3 days preoperatively and then 10,000 IU (150 IU/kg) of subcutaneous rhEPO daily until 3 days postoperatively (6 doses in total). Patients in group C received 1 ml of subcutaneous normal saline daily from 5 days preoperatively to one day preoperatively and then 10,000 IU (150 IU/kg) of subcutaneous rhEPO daily from the day of surgery to 3 days postoperatively (4 doses in total).

**Results:**

One hundred eighty patients were included. On postoperative day one, patients in the group A showed significantly higher Hb level (108.4 ± 11.4 g/L) than group C (103.9 ± 8.8 g/L). Group B (107.8 ± 8.4 g/L) also showed significantly higher Hb level than group C (103.9 ± 8.8 g/L) (*p* < 0.05). On postoperative day 3, no significant difference was found between group B and group C in Hb level (98.7 ± 10.5 and 94.9 ± 8.7 g/L, respectively) (*p* = 0.094), but the Hb level in group A (103.6 ± 11.0 g/L) was still markedly higher than in group B and the Hb level in group A was also markedly higher than in group C. In terms of blood loss, no markedly difference was found in intraoperative blood loss among group A, B and C (78.3 ± 22.4, 84.6 ± 29.1, and 80.3 ± 23.9 ml, respectively) (*p* = 0.381), but on postoperative day one, the mean blood loss in group C (522.4 ± 189.4 ml) was significantly more than group B (371.2 ± 124.6 ml), and group B was also significantly more than group A (284.8 ± 112.9 ml) with 95% confidence interval, and group B had significantly less blood loss than group C (*p* < 0.001). With respect to the total blood loss, the total blood loss in group C (881.6 ± 314.9 ml) was significantly more than group B (642.6 ± 232.9 ml), and group B was also significantly more than group A (514.5 ± 204.6 ml) with 95% confidence interval (Table 2). Only 2 patients in each group received allogeneic blood transfusion and each patient received 2 units of red blood cells, so, the transfusion requirements among the three groups were comparable.

**Conclusions:**

Daily small-dose of subcutaneous rhEPO administered from 5 days before THA could significantly decrease perioperative blood loss and improve postoperative Hb levels, without increasing risks of complications, when compared with the application of rhEPO from 3 days before THA or from the day of surgery. However, surgeons should choose the regimen individually according to different patients’ personal circumstances.

## Background

Total hip arthroplasty (THA) is an effective treatment which provides pain relief and function restoration to patients suffering from end-stage hip disease. Over 500,000 THAs are performed each year in the UK and USA [1]. Approximately 400,000 primary THAs were performed in China in 2015, a number that has been increasing by 25%-30% per year [2]. However, most patients undergoing THA are elderly and combined with a high prevalence (24%) of preoperative anemia, which is widely accepted as a predictive factor of postoperative allogeneic transfusion. Moreover, the procedure is also associated with substantial blood loss leading to a high prevalence (51%) of postoperative anemia which may therefore cause the relatively high rate (45%) of postoperative allogeneic blood transfusion [3]. Allogeneic transfusion carries a substantial risk of transfusion-associated complications requiring additional treatment and increased length of stay [4]. Many blood-saving strategies have been reported, of which recombinant human erythropoietin (rhEPO) has demonstrated effectiveness in reducing hemoglobin (Hb) level drop and blood transfusion requirements according to numerous randomized controlled trials [5, 6, 7, 8, 9].

For now, there exists two main protocols of the perioperative use of rhEPO in patients who are scheduled for total joint arthroplasty (TJA). One is weekly application of large-dose rhEPO for 2–4 weeks before surgery, which is called the long-term regimen [9]. The other one is daily application of small-dose rhEPO from 0–5 days preoperatively to a few days (less than a week) postoperatively, which is called the short-term regimen [5, 6, 7, 8]. However, the weekly protocol has some obvious drawbacks: The long-term of treatment requested the patients to come back to hospital weekly for injections, which would therefore cause inconvenience to the patients and it would also increase the preoperative waiting interval. When encountering patients with poor compliance, it would be even more difficult to implement the treatment. Besides, initial high peak levels from high once weekly doses may be wasted, as erythropoietin receptors on progenitor cells in bone marrow may become saturated; when these receptors are again free for binding, the level of serum erythropoietin will fall [10]. Compared with the weekly protocol, the short-term daily protocol with small amounts of rhEPO could maintain a more constant low but more effective level of serum erythropoietin without causing inconvenience to the patient [10]. Besides, previous studies also reported that repeated administration of rhEPO is more effective in stimulating the reticulocyte response than the weekly large-dose of the same total amount of rhEPO [11].

Cao et al. [6] advocated that a daily dose (150 IU/kg) of rhEPO started from 3 days before arthroplasty is more effective than being applied from the day of surgery in increasing Hb level, reducing blood loss without additional complications, they also concluded that the application of rhEPO since the day of surgery cannot significantly reduce the blood loss or increase the Hb level after arthroplasty compared with no application of rhEPO. On the contrary, Na et al. [8] applied 3000 IU of rhEPO subcutaneously during the operation and the postoperative period, they found that compared with the control (placebo) group, application of rhEPO since the day of surgery could effectively attenuated anemia and decreased transfusion requirements in patients undergoing arthroplasty, which was coincident with the conclusion of Bernabeu-Wittel et al. [5] with the similar rhEPO regimen. Furthermore, Kourtzis et al. [7] advocated the rhEPO regimen that each patient undergoing arthroplasty received 10,000 IU (150 IU/kg) of rhEPO daily subcutaneously for 5 days preoperatively and 3 days postoperatively which is also the current protocol implemented in our center, they found a markedly reduction of 94% in the utilization of allogeneic blood in patients received the rhEPO protocol than the control (placebo) group. Although there exist various regimens, the optimal regimen of short-term daily administration of rhEPO for THA patients are yet to be established.

In this study, a prospective double-blinded randomized placebo-controlled trial comparing three different short-term daily rhEPO-based treatment protocols for blood-saving purpose in THA was conducted. We hypothesized that the daily small-dose of rhEPO started from 5 days before THA which is the current protocol in our center is more effective in blood-saving than starting rhEPO treatment from 3 days preoperatively and the protocol of applying rhEPO from the day of surgery.

### Patients and methods

This double-blinded randomized placebo-controlled trial was registered in the Chinese Clinical Trial Registry (20/09/2019) and the registration number is ChiCTR1900026064. Approval was obtained from the Clinical Trials and Biomedical Ethics Committee of West China Hospital. The study was conducted at the Department of Joint Surgery of West China Hospital, Sichuan University, in accordance with the CONSORT (Consolidated Standards of Reporting Trials) Statement. Written informed consent was obtained from all participants prior to surgery.

#### Patients

We recruited consecutive adult patients (18 to 85 years of age) from September 2019 to May 2020 who were scheduled for primary unilateral THA. Exclusion criteria included a diagnosis other than osteoarthritis or osteonecrosis of the femoral head, a known allergy to rhEPO or ferralia, an Hb level more than 130 g/L or less than 90 g/L, use of spinal anesthesia, a history of a hematopoietic or hemorrhagic disorder, a history of deep venous thrombosis (DVT) or pulmonary embolism (PE), an ongoing anticoagulant treatment with anticoagulant therapy (warfarin or heparin) within 1 week prior to surgery, an ongoing treatment with ferralia and/or rhEPO within 3 months before admission, preoperative hepatic or renal dysfunction and serious cardiac and/or cerebrovascular comorbidities, and refusal of participation.

#### Interventions and surgical procedures

The patients were randomized to 1 of 3 interventions: Patients in group A received 10,000 IU (150 IU/kg) of subcutaneous rhEPO (1 ml) daily from 5 days preoperatively to 3 days postoperatively (9 doses in total); Patients in group B received 1 ml of subcutaneous normal saline daily from 5 days preoperatively to 3 days preoperatively and then 10,000 IU (150 IU/kg) of subcutaneous rhEPO daily until 3 days postoperatively (6 doses in total). Patients in group C received 1 ml of subcutaneous normal saline daily from 5 days preoperatively to one day preoperatively and then 10,000 IU (150 IU/kg) of subcutaneous rhEPO daily from the day of surgery to 3 days postoperatively (4 doses in total). If the Hb level was over 150 g/L, rhEPO was not administered. All patients received a 100 mg (dissolved into 100 ml of normal saline) dose of intravenous ferric carboxymaltose, which has been widely accepted as an effective partner with rhEPO for the blood-saving purpose in patients undergoing arthroplasty [8]. The placebo (normal saline) has the same appearance (colorless and clear liquid) as rhEPO.

All the THAs were performed by the same surgical team (One main surgeon and three assistants) using a posterolateral approach and a single brand of cementless acetabular and femoral components (DePuy Synthes) under general anesthesia. No autologous blood transfusion or postoperative drain were used(12).

#### Thromboembolism prophylaxis and transfusion protocol

All the patients received thromboembolic prophylaxis according to a standardized protocol at our institution, which is a combination of physical prophylaxis and chemoprophylaxis [12]. The patients received physical prophylaxis by means of an intermittent inflatable pump system on the day after surgery. As for the chemoprophylaxis, a half-dose (2000 IU in 0.2 mL) of low-molecular-weight heparin was given to patients subcutaneously 6 h postoperatively and a full dose (4000 IU in 0.4 mL) was repeated at 24-h intervals subsequently until discharge. After discharge, all patients routinely received 10 mg rivaroxaban for 10 days. During hospitalization, the patients were examined daily for any clinical symptoms of DVT. Ultrasound examinations were also performed routinely at preoperatively, discharge, and the 2-week follow-up. A chest contrast-enhanced spiral computed tomography was performed immediately for any clinical signs of PE [12].

A standardized blood-transfusion protocol of the National Ministry of Health was followed for all patients: Blood transfusion was indicated for any patients with a Hb level of < 70 g/L, or a Hb level between 70 and 100 g/L but with symptomatic anemia (severe mental status changes, palpitations, and/or pallor) [12].

#### Outcome measurements

The primary outcomes included the Hb levels of the three groups at different time points, the rate and amount of allogeneic transfusion, and intraoperative and total blood loss. Secondary outcomes included the reticulocyte count, complications and patient satisfaction level. All the blood samples were obtained before each injection of rhEPO at 7 time points (on the preoperative day 5, 3, and 1, and on the postoperative day 1, 3, 14, and 21), except the operation day, on which blood samples were obtained both at one hour preoperatively and immediately after surgery. Blood loss was calculated from the change in hematocrit using the formula of Nadler et al. and Gross plus the volume transfused [13, 14]. Besides, all the complications, including DVT, PE and other adverse effects related to rhEPO (nausea, fever, headache, myalgia, etc.) were also assessed during the first 3 weeks postoperatively. Moreover, patient satisfaction level was evaluated with a simple satisfaction questionnaire at the time of discharge (We used the table of satisfaction with 6 options: Extremely satisfied, very satisfied, Somewhat satisfied, Neither satisfied nor dissatisfied, Somewhat dissatisfied and Very dissatisfied) [15].

#### Randomization and blinding

A random allocation sequence was computer-generated and concealed in consecutively numbered, opaque, sealed envelopes by a research statistician not involved in the data analysis. One of 2 experienced surgeons enrolled the patients, and the other one recorded basic detail. The envelope was opened after the enrollment of patients, and the study medication and placebo were prepared by a dedicated nurse not involved with patient care or outcome measurement. Patients, surgeons, anesthesiologists, care providers, and data collectors were all blinded to the allocation sequence.

#### Statistical analysis and sample size

The sample size was determined in relation to the difference in the postoperative decrease in Hb level among the 3 study groups using G*Power Version 3.1.7 (Franz Faul; UniKiel, Germany) software. On the basis of our preliminary data of 60 patients who underwent unilateral primary total hip arthroplasty and were assessed for the same measure, the mean decrease in Hb level (and standard deviation) was 41.5 ± 6.39 g/dL. A reduction of 10% (4 g/dL) in the decrease in Hb level in the experimental group (daily dose of 10,000 IU from 5 days preoperatively to 3 days postoperatively) compared with the control group (placebo only) was recorded as a clinically meaningful difference. On the basis of this information, 60 patients were required in each arm, with an alpha of 5%, power of 85%, and an anticipated 20% dropout rate.

Distributions of demographic data, baseline data, surgical characteristics, and primary and secondary outcomes were assessed using measures of central tendency (mean, standard deviation) for quantitative variables and with percentages for qualitative variables. Continuous variables were compared using one-way analysis of variance. When significant differences were detected, the 3 experimental groups were compared with each other using the Tukey's post hoc honest significant difference test. Categorical variables were compared using the chi-squared or Fisher's exact tests. All data analyses were performed using SPSS for Windows, Version 19.0 (SPSS Inc, Chicago, IL). Significance was set at *P* < 0.05.

## Result

**Fig. 1:**
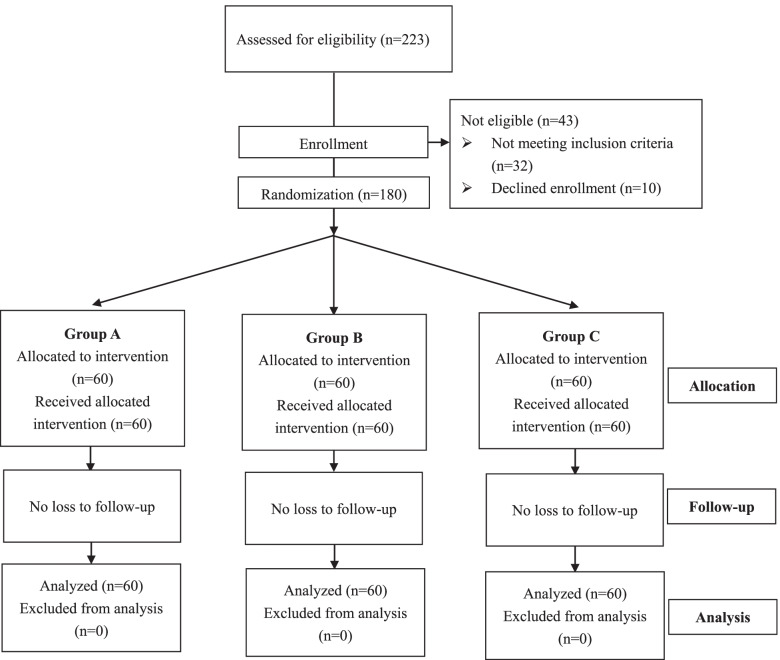
Patients flow chart

From September 2019 to May 2020, 223 patients scheduled for primary unilateral THA were evaluated for eligibility, of which a total of 43 patients were excluded (32 did not meet the inclusion criteria and 10 declined to participate). Finally, the rest 180 patients were randomly assigned to 1 of the 3 groups (60 patients in each group) (Fig. 1). During the follow-up period of postoperative 3 weeks, no patient was lost or excluded for any reason. The mean hospitalization day in all the groups are all 6.2 days. Patient demographic and preoperative characteristics in all the three groups were comparable (Table 1).

**Table 1:** Baseline demographic and clinical characteristics

Variable	Group A (*n* = 60)	Group B (*n* = 60)	Group C (*n* = 60)	*P* Value
Demographic characteristic				
Age	66.40 ± 9.82	63.21 ± 10.82	64.38 ± 9.14	0.2093
Female (no. [%] of patients)	39 (65.0%)	34 (56.7%)	36 (60.0%)	0.643
Height	1.60 ± 0.07	1.62 ± 0.06	1.61 ± 0.07	0.2636
Weight	64.12 ± 9.84	65.73 ± 10.15	66.31 ± 9.91	0.4611
BMI	26.11 ± 4.02	25.81 ± 3.87	26.52 ± 3.41	0.5868
Operated side (no. of patients)				0.420
Left	31	26	33	
Right	29	34	27	
Diagnosis (no. of patients)				0.849
ONFH	22	25	23	
OA	38	35	37	
ASA class (no. of patients)				0.734
I	11	13	12	
II	43	40	41	
III	6	7	7	
Preoperative laboratory values				
Hemoglobin (g/L)	120.7 ± 9.3	119.9 ± 10.4	122.4 ± 9.2	0.3509
Reticulocyte (× 10^12/L)	0.0676 ± 0.031	0.0714 ± 0.028	0.0663 ± 0.025	0.5929

*BMI* body mass index, *ONFH* osteonecrosis of femoral head, *OA* osteoarthritis *ASA* American Society of Anesthesiologists

The *p* value represents the result of one-way analysis of variance for independent means for continuous variables or the chi-square test for independent proportions among the 3 groups

### Primary outcome

**Fig. 2:**
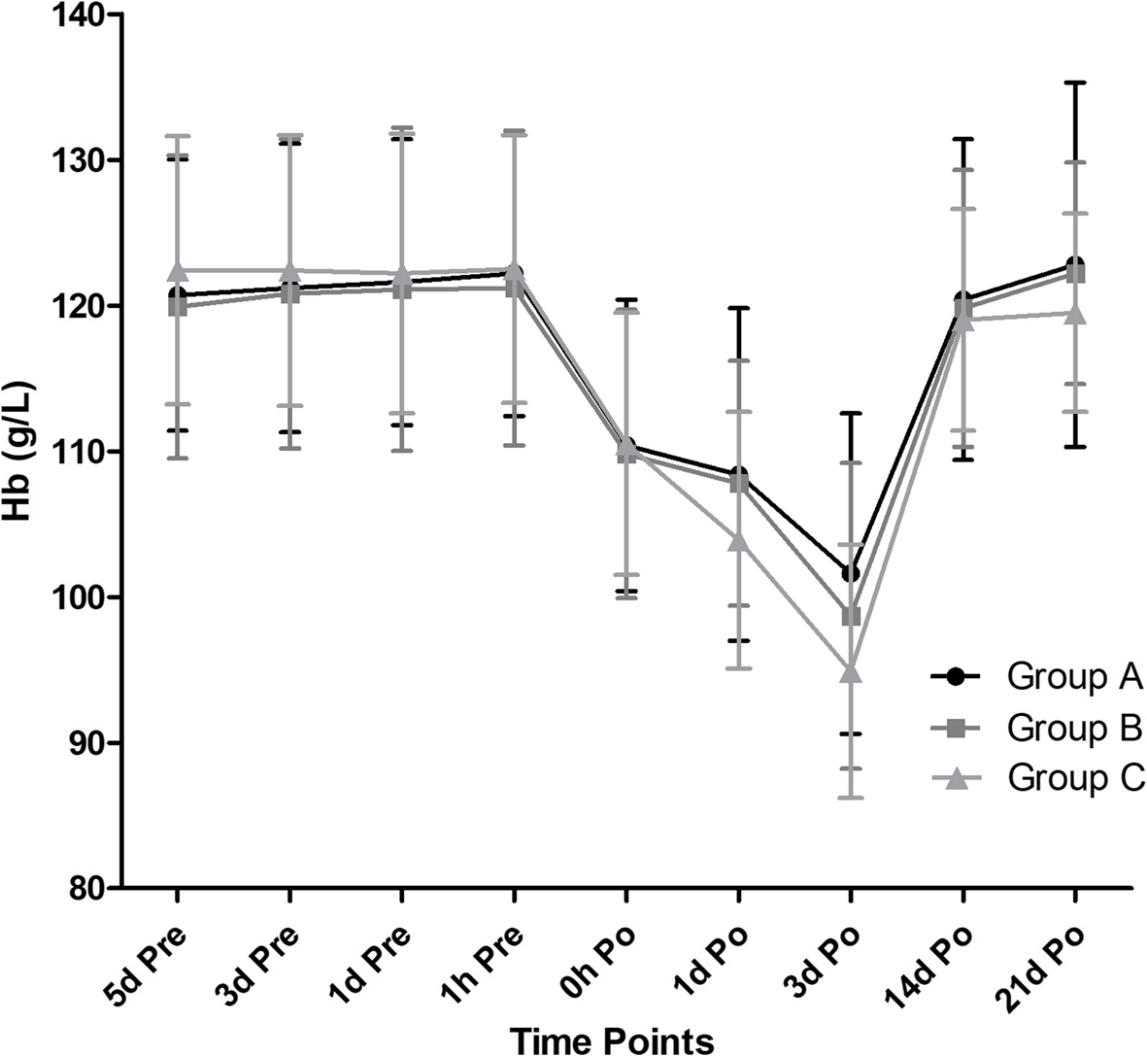
Change in mean Hb level from baseline during the study

On postoperative day one, patients in the group A showed significantly higher Hb level (108.4 ± 11.4 g/L) than group C (103.9 ± 8.8 g/L) (*p* < 0.05), the Hb level in group B (107.8 ± 8.4 g/L) was also markedly higher than in group C (*p* = 0.045). However, on postoperative day 3, no significant difference was found between group B and C in Hb level (98.7 ± 10.5 and 94.9 ± 8.7 g/L, respectively) (*p* = 0.094), even if group A (103.6 ± 11.0 g/L) was higher than group B and group A was also higher than C with 95% confidence interval. No difference was found among the three groups at other time points. (Fig. 2).

In terms of blood loss, no markedly difference was found in intraoperative blood loss among group A, B and C (78.3 ± 22.4, 84.6 ± 29.1, and 80.3 ± 23.9 ml, respectively) (*p* = 0.381), but on postoperative day one, the mean blood loss in group C (522.4 ± 189.4 ml) was significantly more than group B (371.2 ± 124.6 ml), and group B was also significantly more than group A (284.8 ± 112.9 ml) with 95% confidence interval. With respect to the total blood loss, the total blood loss in group C (881.6 ± 314.9 ml) was significantly more than group B (642.6 ± 232.9 ml), and group B was also significantly more than group A (514.5 ± 204.6 ml) with 95% confidence interval (Table 2). Only 2 patients in each group received allogeneic blood transfusion and each patient received 2 units of red blood cells, so, the transfusion requirements among the three groups were comparable.

**Table 2:** Blood loss during the postoperative period

Variable	Group A (*n* = 60)	Group B (*n* = 60)	Group C (*n* = 60)	Pairwise Comparison (*P* value)			*P* Value
				Group A vs B	Group A vs C	Group B vs C	
Intraoperative blood loss	78.3 ± 22.4	84.6 ± 29.1	80.3 ± 23.9	0.3621	0.9019	0.6215	0.3805
Blood loss till postoperative day 1	284.8 ± 112.9	371.2 ± 124.6	522.4 ± 189.4	< 0.01^a^	< 0.001^a^	< 0.001^a^	< 0.001^a^
Total blood loss	514.5 ± 204.6	642.6 ± 232.9	881.6 ± 314.9	0.0179^a^	< 0.001^a^	< 0.001^a^	< 0.001^a^

The *p* value represents the result of one-way analysis of variance for independent means for continuous variables

^a^means significant difference

### Secondary outcome

**Fig. 3:**
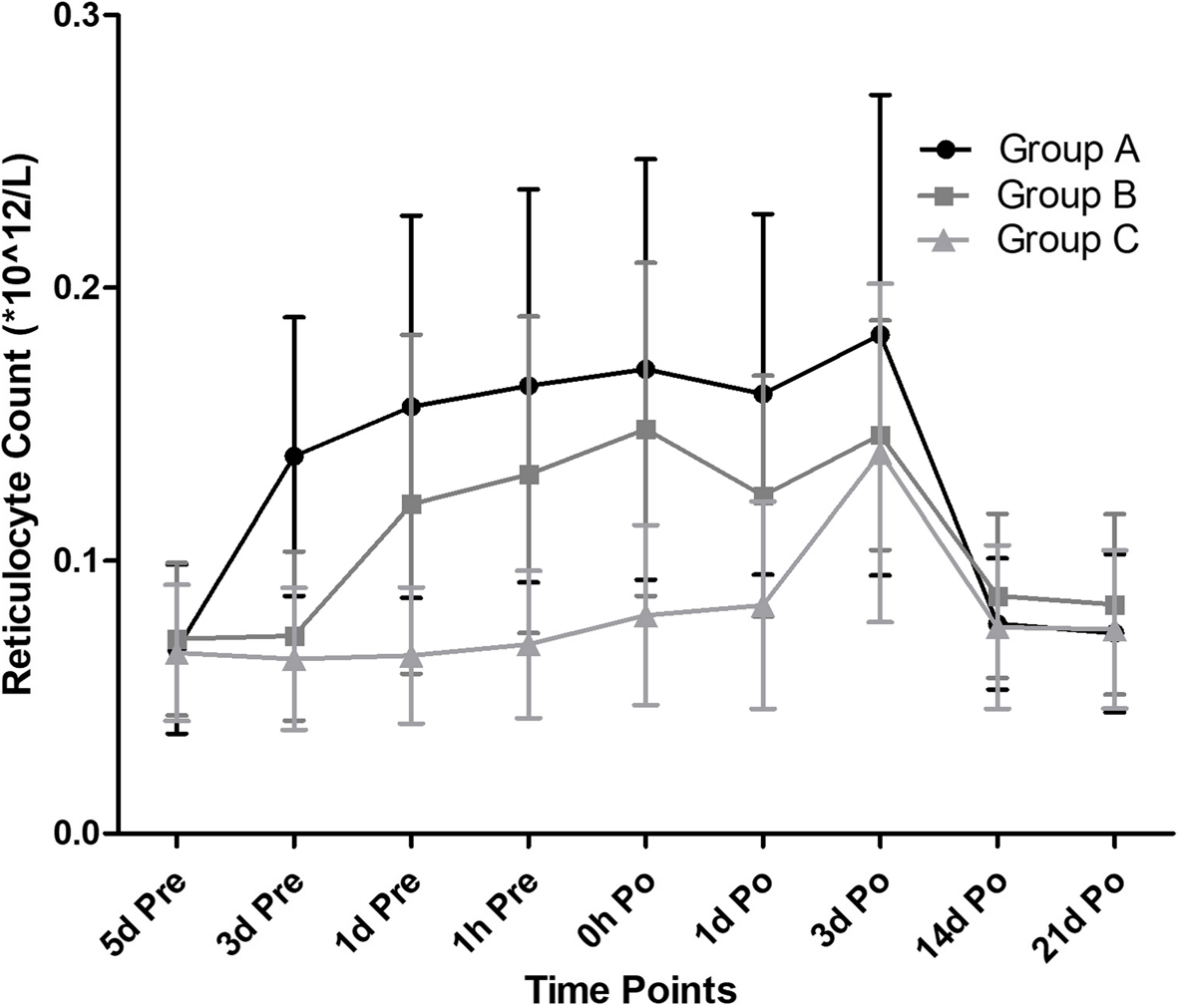
Change in mean reticulocyte count from baseline during the study

The reticulocyte count increased quickly after application of rhEPO. From 3 days preoperatively to 3 days postoperatively, reticulocyte counts in group A was always higher than group B, also the reticulocyte counts in group A was always higher than group C (*p* < 0.01). Besides, from the day before surgery to the day after surgery, reticulocyte counts in group B were always higher than in group C (*p* < 0.001) (Fig. 3). In terms of complications, no case of DVT occurred in any group, only a few cases of asymptomatic intermuscular vein thrombosis (IMVT) were observed in group A (3, 5.0%), B (3, 5.0%) and C (2, 3.3%) without markedly difference among groups (*P* = 0.877). The all three regimens of rhEPO in this study were generally well tolerated, with few adverse events: nausea in 9, pyrexia in 7, headache in 4, muscle pain in 4 patients but without markedly difference among all the three groups (*P* > 0.05) (Table 3). No case of death or readmission within postoperative 21 days occurred. Moreover, most patients were very satisfied with the treatment they received. (Table 3).

**Table 3:** Complications

Variable	Group A (*n* = 60)	Group B (*n* = 60)	Group (*n* = 60)	*P* Value
Complications				
DVT	0	0	0	NA
IMVT	3	3	2	0.877
Nausea	3	2	4	0.704
Pyrexia	3	2	2	0.862
Headache	1	2	1	0.774
Muscle pain	2	1	1	0.774

*NA* not applicable, *DVT* deep vein thrombosis, *IMVT* intermuscular vein thrombosis

The *p* value represents the result of the chi-squared test or Fisher's exact tests for categorical variables among the 3 groups

## Discussion

There is general agreement of the effective function of rhEPO in reducing transfusion requirements and accelerating the recovery of Hb level after THA [5, 6, 7, 8, 9, 10]. Despite the common application of rhEPO in total joint arthroplasty during the last decade, with benefits including attenuated Hb drop, decreased blood loss, and reduced transfusion requirement, no final consensus has been reached with respect to the optimal regimen, which is yet to be investigated.

Prior studies mostly focused on the application of rhEPO in TJA patients started from 2–4 weeks before surgery and each dose were large (300–600 IU/kg), and the protocol was generally accepted as the standard regimen [9, 16, 17]. However, the long-term of preoperative treatment requested the patients to come back to hospital weekly for injections, which would not only cause inconvenience to the patients but also increase the preoperative waiting interval. Contrary to the studies supporting the long-term and high-dose protocol, some investigators reported that more frequent perioperative application of small-dose rhEPO could be more efficacious. Cody et al. [10] suggested that weekly high dose of rhEPO may be wasted for the limited erythropoietin receptors on progenitor cells in marrow which is easy to be saturated. When the receptors are saturated, no amount of rhEPO is effective until the receptors are again free for binding, but by then, the level of serum rhEPO would drop. So, frequent application of small-dose rhEPO could maintain a more constant low but more effective level of serum rhEPO. Similarly, Cheung et al. [11] found that repeated application of rhEPO is more effective in stimulating the reticulocyte response than the single dose even if the total amount of rhEPO given to patients are the same. However, even if being applied in frequent small-dose, the rhEPO still has many regimens without a consensus.

In this study, we summarized and compared the main three types of perioperative rhEPO regimens, and found: Hb levels in the three groups didn’t show difference until the postoperative day one, when the Hb level in group A was significantly higher than in group C, in the meantime, Hb level in group B was also markedly higher than in group C. In terms of transfusion requirement, no difference in either transfusion rates or amounts was found among all the three regimens, even if the total blood loss in group A was the most in the three groups. Few treatment-related adverse events occurred perioperatively, indicating the three regimens of rhEPO in this study were generally well tolerated, and no markedly difference in complication rates were observed among the three groups.

### Primary outcomes

#### Hb levels

It was reported that rhEPO could exert its effect of attenuating postoperative Hb drop soon after administration [8], and the Hb peaked on approximately 8–10 days subsequently [18]. In this study, on postoperative day 1, patients in group A showed significantly higher Hb level than those in group C, the Hb level in group B was also markedly higher than group C, which indicated that the application of rhEPO started from 5 and 2 days preoperatively was better than the day of surgery. On postoperative day 3, when the Hb was the lowest during the postoperative period of THA and the transfusion also happened most likely in this period [19], patients in group A showed significantly higher Hb level than group B, also group A showed significantly higher Hb level than group C with 95% confidence interval, which indicated the Hb drop of group A was the least. The application of rhEPO in group A started 5 days before surgery which means that it has been 8 days until 3 days postoperatively when the erythropoiesis estimated by the rhEPO reached the peak, which is coincident with the prior studies. However, it was reported by Goodnough et al. [20] that for patients subjected to a relatively large amount of blood loss, the endogenous erythropoietin response would be very substantial. Besides, in one clinical trial conducted by Goodnough et al. [21], a linear–logarithmic relationship was demonstrated between change in Hb level and endogenous erythropoietin response, which meant that the more Hb drop, the stronger the endogenous erythropoietin response was, even if the effect is small. In this study, comparing postoperative day 1 and 3, the Hb level between group B and C became comparable. It was the sharp drop of Hb in group C that led to a sharp increase of the endogenous erythropoietin response, which could therefore stimulate erythropoiesis quickly, while the drop of Hb level in group B was not so large that the exogenous rhEPO in group B still needed more time to stimulate erythropoiesis and promote the Hb level after surgery.

#### Blood loss and transfusion

It was reported that no matter under which kind of rhEPO regimen, the intraoperative blood loss would not be decreased: Kourtzis et al. [7] applied rhEPO from preoperative 5 days and recorded the comparable intraoperative blood loss between study and control group. Similarly, Cao et al. [6] recorded the intraoperative blood loss between two rhEPO regimens, one started from 3 days preoperatively and the other one from the day of surgery, and they found comparable intraoperative blood loss between the two groups. The trials above all indicated that the intraoperative blood loss is a parameter that has nothing to do with the application of rhEPO, which was also coincident with our study. In terms of total blood loss, we calculated the blood loss by using the Gross formula with the hematocrit (HCT) and the blood volume (calculated by using the weight and height of patients) [13]. It was reported by Ma et al. [22] that on postoperative day 3, the patients undergoing TJA had the lowest HCT level of the whole perioperative period, so the blood loss calculated by using the HCT tested preoperatively and 3 days postoperatively could indirectly reflect the blood loss of patients. Even if the data of blood loss was a calculated value, it could still be used to compare between patients to find out relatively more or less blood loss. So, in this study, we firstly recorded the perioperative HCT and found that the HCT on 3 days postoperatively was the lowest, then we compared the total blood loss by using the calculated blood loss (with the HCT which was tested preoperatively and 3 days postoperatively) and found that in terms of total blood loss, group A was significantly less than group B, in the meanwhile, group B was also significantly less than group C, which suggested that the application of rhEPO 5 days before surgery could achieve the least total blood loss.

With respect to transfusion requirements, prior studies all reported that application of rhEPO, no matter under which regimen, would decrease the requirement of allogeneic transfusion [5, 6, 7, 8, 9, 10, 16, 17]. However, in this study, only 2 patients in each group received allogeneic transfusion of 2 units of red blood cells. The very low transfusion requirements might due to the relatively little blood loss of patients in our center. The perioperative use of tranexamic acid [23], the intraoperative controlled hypotension as well as the application of rhEPO combined with ferralia all accounted for the little blood loss and the low transfusion rate.

### Secondary outcomes

#### Reticulocyte count

Because reticulocytes are normally released from the marrow 18 to 36 h before their final maturation into erythrocytes, they provide a real-time assessment of the functional state of erythropoiesis [20]. Ait-Oudhia et al. [18] discovered that blood reticulocyte count peaked on 72 h to day 5 after application of rhEPO. In this study, on the day before surgery, the reticulocyte count in group A and B were markedly larger than in group C, which was consistent with the prior studies. In group C, however, the application of rhEPO started from the day of surgery which was relatively late. So, we found that on postoperative day one the reticulocyte count in group C didn’t increase as much as those in group A and group B, which not only reflected that the erythropoiesis effect stimulated by rhEPO has not peaked yet but also demonstrated that the endogenous erythropoiesis could not be quickly mobilized when meeting blood loss or acute anemia postoperatively. However, on postoperative day 3 the reticulocyte count in group C quickly caught up with the other two groups, which might indicate the endogenous erythropoiesis come into play, the more the blood loss the stronger the effect become. Finally on postoperative day 14 and 21, no markedly difference was observed in reticulocyte count among the three groups, indicating that the marrow hematopoietic phase mobilized by perioperative application of rhEPO was over.

#### Complications

In theory, rhEPO could cause increased platelet count, enhanced blood viscosity, leading to hypercoagulability, thus increasing the risk of thrombosis [24, 25]. Some investigators reported that patients after THA and TKA are naturally at high risk for developing DVT and the application of rhEPO might increase such risk, which limited the application of rhEPO to some extent [24, 25]. However, in this study, no case of DVT were found in all the three groups. Only a few cases of asymptomatic IMVT were observed without markedly difference among groups. It was reported that no significant correlation was found between the incidence of postoperative PE and IMVT, and no special treatment to postoperative IMVT was needed [26]. Besides, the three regimens of erythropoietin in this study were generally well tolerated, with few treatment-related adverse events such as nausea, pyrexia and headache and no difference were found in rates of different adverse events among three groups. Most patients were satisfied with the treatment they received during the whole hospitalization and follow-up phases.

### Limitation and advantage

Although this study was carefully designed, several limitations still exist. First, the sample size was calculated according to the change in Hb level, which would be therefore not sufficient to identify a significant difference in other parameters like the transfusion rates and different complication rates. Second, we did not record the platelet count or the indexes related to coagulation such as D-dimer or Fibrin/Fibrinogen Degradation Products (FDP), so it might be inaccurate to judge that the three different regimens of rhEPO had no difference in the influence on patients’ risk of DVT by simply recorded the rates of DVT or IMVT. The comparable rates of DVT among the three groups might also partly due to the anticoagulant therapy implemented in this study which has been proven to be efficacious and safe in our previous study [12].

It was reported by prior studies that the application of rhEPO perioperatively could relatively increase the medical costs [27]. In this study, although we did not collect data with respect to the costs or conduct the cost-effective analysis, we still concerned the potential financial burden and feasibility of establishing such protocols. Even though the regimen of small-dose of subcutaneous rhEPO daily from 5 days preoperatively to 3 days postoperatively for patients scheduled for THA (regimen A) could reduce the blood loss of patients, it needs patients coming to hospital for injection more frequently than other regimens, which not only increases the transportation fee, but also increases the invisible risk of patients (knocked down by something or slip on the ground). Especially for some patients with severe knee/hip pain, the more frequently they go outdoors, the more pain they would suffer. Besides, the cost of the rhEPO in regimen A is of course higher than the others for the more amount of use. So, when choosing rhEPO protocols, surgeons should consider individually according to different patients’ personal circumstances.

## Conclusions

Daily small-dose of subcutaneous rhEPO administered from 5 days before THA could significantly decrease perioperative blood loss and improve postoperative Hb levels, without increasing risks of complications, when compared with the application of rhEPO from 3 days before THA or from the day of surgery. However, surgeons should choose the regimen individually according to different patients’ personal circumstances.

## Data Availability

Data used and analyzed in this study are available from the corresponding author on reasonable request.

## Abbreviations

rhEPO: Recombinant Human Erythropoietin
THA: Total hip arthroplasty
BMI: Body mass index
ONFH: Osteonecrosis of femoral head
OA: Osteoarthritis
ASA: American Society of Anesthesiologists
Hb: Hemoglobin
Ret: Reticulocyte
TJA: Total joint arthroplasty
DVT: Deep venous thrombosis
PE: Pulmonary embolism
IMVT: Intermuscular vein thrombosis
